# Changes in DNA methylation assessed by genomic bisulfite sequencing suggest a role for DNA methylation in cotton fruiting branch development

**DOI:** 10.7717/peerj.4945

**Published:** 2018-06-14

**Authors:** Quan Sun, Jing Qiao, Sai Zhang, Shibin He, Yuzhen Shi, Youlu Yuan, Xiao Zhang, Yingfan Cai

**Affiliations:** 1 Henan University, State Key Laboratory of Cotton Biology, Henan Key Laboratory of Plant Stress Biology, School of Life Sciences, School of Computer and Information Engineering, Kaifeng, Henan, China; 2 Chongqing University of Posts and Telecommunications, College of Bioinformation, ChongQing, China; 3 Cotton Institute, Chinese Academy of Agricultural Sciences, Anyang, Henan, China

**Keywords:** DNA methylation, Bisulfite sequencing, Fruiting branch, Development

## Abstract

Cotton plant architecture, including fruit branch formation and flowering pattern, influences plant light exploitation, cotton yield and planting cost. DNA methylation has been widely observed at different developmental stages in both plants and animals and is associated with regulation of gene expression, chromatin remodelling, genome protection and other functions. Here, we investigated the global epigenetic reprogramming during the development of fruiting branches and floral buds at three developmental stages: the seedling stage, the pre-squaring stage and the squaring stage. We first identified 22 cotton genes which potentially encode DNA methyltransferases and demethylases. Among them, the homologous genes of *CMT*, *DRM2* and *MET1* were upregulated at pre-squaring and squaring stages, suggesting that DNA methylation is involved in the development of floral buds and fruit branches. Although the global methylation at all of three developmental stages was not changed, the CHG-type methylation of non-expressed genes was higher than those of expressed genes. In addition, we found that the expression of the homologous genes of the key circadian rhythm regulators, including *CRY*, *LHY* and *CO*, was associated with changes of DNA methylation at three developmental stages.

## Introduction

DNA methylation is an epigenetic modification that is inherited by the next generation but does not involve alterations to DNA sequences in both plants and animals (Cubas, Vincent & Coen, 1999; Feng, Jacobsen & Reik, 2010; Manning et al., 2006; Patterson, Thorpe & Chandler, 1993; Xiang et al., 2010; Zhang et al., 2006). DNA methylation plays a key role in many biological processes, including imprinting, transposon silencing, flower and seed development, fruit ripening and stress responses (Dowen et al., 2012; Jullien et al., 2006; Xiao et al., 2006; Xing et al., 2015; Yang et al., 2015; Zhong et al., 2013). In plants, DNA methylation generally occurs in three nucleotide sequence contexts: CG, CHG and CHH (H indicates C, T or A).

CG methylation is generated by the conserved DNA methyltransferase METHYLTRANSFERASE1 (MET1), and CHG methylation is produced by the plant-specific DNA methyltransferase CHROMOMETHYLASE3 (CMT3), while de novo CHH methylation is established through a 24-nucleotide small interfering RNA (siRNA)-dependent DNA methylation (RdDM) pathway and terminated by the DNA methyltransferases DOMAINS REARRANGED METHYLTRANSFERASE1 (DRM1) and DRM2 in plants (Law & Jacobsen, 2010; Stroud et al., 2014; Zemach et al., 2013). Additionally, CHH methylation is established by CMT2 and the DNA methyltransferase-like protein Dnmt2, but its function in regulating DNA methylation is largely unknown (Zemach et al., 2013). In *Arabidopsis*, DNA demethylation primarily depends upon the activity of the DNA glycosylase DEMETER (DME) (Choi et al., 2002). The other DNA glycosylases such as REPRESSOR OF SILENCING1 (ROS1), DEMETER-LIKE2 (DML2), and DML3 are also involved in the removal of 5-methylated cytosine in *Arabidopsis* (Gong et al., 2002).

Cotton is one of the most important economic crops due to its natural textile fibre (Paterson et al., 2012). Cotton architecture is primarily determined by shoot branching and flowering patterns that directly influence plant light exploitation and cotton yield (Chen et al., 2015; Reinhardt & Kuhlemeier, 2002; Sakamoto & Matsuoka, 2004). In cotton, floral bud formation implies the beginning of reproductive growth following vegetative growth, and most cultivated cotton varieties produce lateral branches from the leaf axils. Floral bud bursting and fruit branching are major events in the development of cotton architecture and are among the most important productivity-related agronomic traits in cotton breeding and cultivation (Guo et al., 2009; Li et al., 2012). Elucidating the molecular mechanisms underlying the transition from vegetative to reproductive growth will be useful for optimizing plant architecture and therefore results in higher yields and lower planting costs.

In recent years, a number of studies have identified the key genes involved in the floral transition, branching, shoot apical meristem development, and branch angle formation. For instance, Branched1 play a central role in the strigolactone pathway and branching and Tiller Angle Control 1 is involved in determining plant architecture in rice and *Arabidopsis* (Busch et al., 2011; Niwa et al., 2013; Yu et al., 2007). McGarry et al. observed that overexpression of Arabidopsis *FLOWERING LOCUS T* in cotton affected plant architecture (Li et al., 2012; McGarry & Ayre, 2012a, 2012b; McGarry, Prewitt & Ayre, 2013; McGarry et al., 2016; Yu et al., 2014). However, little is known about the molecular mechanisms of cotton architecture development. In our previous study, high-throughput next-generation sequencing technology was used to simultaneously measure mRNA and miRNA expression profiles during fruit branch and floral bud development in cotton and identified several genes potentially related to the floral transition and branching development (Sun et al., 2016). In the apple, Guo et al. observed significant changes in 24 nucleotide sRNAs (RdDM pathway), suggesting that the epigenetic modifications is coupled with the floral transition (Guo et al., 2017). In *Arabidopsis*, DNA methylation may be associated with flower development and tetrahydrofolate controls flowering time through adjusting DNA methylation-regulated gene expression (Wang et al., 2017; Yang et al., 2015). However, whether the cotton floral transition and branching development are regulated by DNA methylation remains unknown.

In this study, DNA methylation was investigated in the floral transition and branch development of cotton using whole-genome bisulfite sequencing (BS-seq). We analysed the changes of DNA methylation at three stages during floral bud development: the seedling stage, the pre-squaring stage and the squaring stage. We constructed dynamic maps in combination with previously published mRNA and miRNA data (derived from the same cotton samples as used in this study) to analyse the expression profile of DNA methylation-related gene. Our research provides a method to exploit the possible role of DNA methylation during the cotton floral transition and branching development.

## Materials and Methods

### Plant material

The cotton varieties Huazhong 94-3130 (*G. hirsutum L.*, normal branch) were obtained from the Cotton Institute of the Chinese Academy of Agricultural Sciences of China and were grown in field (Kaifeng, China). Shoot apices (approximately 10 mm) were collected and stored immediately in liquid nitrogen after washing with water. The samples at three stages, including the seedling stage with two leaves (Gh1nm), the pre-squaring stage with six and seven leaves (Gh2nm) and without any visible triangular floral bud (only floral bud and fruiting branch formation), and the squaring stage with 8–10 leaves (Gh3nm) with visible triangular floral buds in the field, were used to construct libraries (Sun et al., 2016). The indicated leaves refer to visual, expanded true leaves in the cotton field.

### Identification of DNA methylation-related genes in cotton

The amino acid sequences of *Arabidopsis* DNA methylation-related genes were obtained from the Arabidopsis Information Resource (TAIR) database. Then, Arabidopsis protein sequences were used as queries in local BLAST software against the cotton protein database. The threshold was set at expectation value (*E*-value) 10^−10^ (version: 2.2.6). The hit amino acid sequences were extracted by Perl script based on their ID and were further analysed by SMART network (http://smart.embl-heidelberg.de/) for the conserved domain.

### DNA extraction and BS-Seq

Each sample is a mixture of three or more plant tissues and the DNA was isolated using a cetyltrimethylammonium bromide method (CTAB) plant genomic kit (Aidlab, Peking, China). The gDNA was checked for integrity by agarose gel electrophoresis and the concentration was measured using a non-ultraviolet method. The DNA was fragmented using a Diagenode sonicator to a mean size of 100–300 bp, followed by DNA-end repair, 3’-dA overhangs and the ligation of methylated sequencing adaptors according to the manufacturer’s instructions (Illumina, San Diego, CA, USA). DNA samples were bisulfite-treated using the ZYMO EZ DNA Methylation-Gold Kit (ZYMO Research, Irvine, CA, USA). The bisulfite-converted DNA was then amplified by PCR and purified after desalting and size selection. Quantified and converted DNA libraries were loaded onto the Illumina HiSeq2000 platform and paired-end sequencing was performed to generate paired-end 125-bp reads for each library. The sequencing was carried out without biological replicate.

### BS-seq data analysis

After removing low-quality reads, clean data were mapped to the cotton reference genome (http://mascotton.njau.edu.cn) (Zhang et al., 2015) using BSMAP software (version: 2.9) (Xi & Li, 2009), which allows up to 8% mismatch per read. Then, we used a custom Perl script to calculate methylation levels based on the cytosine percentage in a given position. The methylation profiles for flanking 2-kb regions and the gene body (or transposable element (TEs)) were plotted based on the average methylation levels for each 100-bp interval. Additionally, we identified different methylation regions **(**DMRs) for each sequence context (CG, CHG and CHH) between the three tissue samples using the tDMR package (Rakyan et al., 2008) with the following stringent criteria: (a) at least five methylated cytosine sites in at least one sample; (b) coverage by more than ten reads; (c) a distance between adjacent methylated sites was <200 bp; (d) a length of the region was between 40 bp and 10,000 bp; and (e) a difference in methylation levels was at least two with a Pearson’s χ2 test value of *p* ≤ 0.05. Putative DMRs overlapping at adjacent 2-kb (upstream or downstream) or body regions of genes or TEs were sorted out for further study.

### The expression profile of genes related to DNA methylation

Published gene expression data (GEO accession no. GSE69025) of the same samples as used in this study were further analysed (Sun et al., 2016). Gene expression profile was computed using the TopHat software package and differentially expressed genes (DEGs) were screened using the edge R package (log2|Fold Change| > 1 and FDR < 0.05). After the calculated *p*-value was corrected by Bonferroni and *q*-value was corrected by multiple examination, Gene ontology analysis and Kyoto Encyclopedia of Genes and Genomes (KEGG) enrichment analysis were used for predicting candidate gene function. The threshold value *p*-value ≤ 0.05 and *q*-value ≤ 0.05 were used for estimate statistical significance, respectively. Patmatch_v.2 software is used to make the complementary pairing of small RNAs and target genes. The different expression siRNA with its target genes that have contrary expression change trend with siRNA were used for regulatory networks diagraph analysis on OmicShare tools, a free online platform for data analysis (http://www.omicshare.com/tools).

### DMR validation

To confirm whether DNA methylation levels were similar to the result of BS-seq, we choose the Gh_A10G0138 genome sequence between 1161268 bp and 1161628 bp on chrA10 in sample Gh1nm for validation. The genomic DNA was subjected to bisulfite treatment using the EZ DNA Methylation-Lightning Kit (ZYMO Research, Irvine, CA, USA) according to the manufacturer’s instructions. The bisulfite-converted DNA was PCR-amplified with ExTaq DNA Polymerase (Takara, Dalian, Shandong, China). The primers were designed using the website tool at http://katahdin.mssm.edu/kismeth/primer_design.pl (Gruntman et al., 2008). PCR products were amplified with the primer pair 5’-TAGATTTGGGTGTGGTTGGATG-3’ and 5’-CTTTARAACATTRCCTAACAAAACA-3.’ PCR products were cloned into the pMD18-T vector and 15 clones were randomly selected for sequencing. The final sequencing results were analysed using QUMA (http://quma.cdb.riken.jp/) (Kumaki, Oda & Okano, 2008).

## Results

### Identification and expression profile of DNA methylation-related genes in cotton

**Figure 1:**
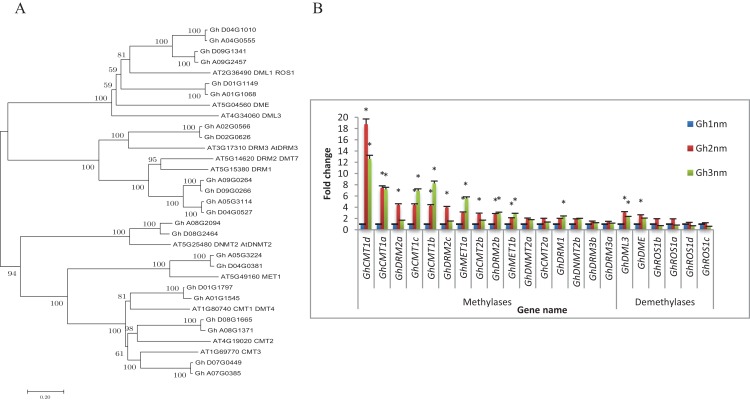
Evolutionary relationships of taxa and expression profiles of DNA methyltransferase and demethylase genes in three stages of cotton. (A) The evolutionary relationships of taxa. The evolutionary history was inferred using the Neighbor-Joining method. The bootstrap consensus tree inferred from 1,000 replicates is taken to represent the evolutionary history of the taxa analysed. (B) Expression profiles of DNA methyltransferase and demethylase genes by RNA-seq. Note: *Means significant with FDR < 0.05 (the *p*-value value after FDR correction) and |log2FoldChange| >1. Error bars mean ± SD of three replicates.

The balance between DNA methylation and DNA demethylation may mediate specific DNA methylation during the development of cotton tissue. To study the DNA methylation modifications that occur during cotton branch development, cotton DNA methyltransferases and demethylases were identified and expression profiles of these genes were constructed based on previously published RNA-seq data (Sun et al., 2016). As shown in Table 1, we identified a total of 22 genes encoding 16 DNA methyltransferases and six demethylases in the cotton genome based on the protein sequence homology of *Arabidopsis* DNA methyltransferases (including *MET1*, *DRM*, *CMT*, and *Dnmt2*) and DNA demethylases (including *DME*, *ROS1* and *DML3*) (Fig. 1A; Fig. S1).

**Table 1:** Identification of genes encoding DNA methyltransferases and demethylases in cotton.

Gene ID	Name	Location	Protein length	Identity (%)	Homologs	Annotation
**DNA methyltransferases**						
Gh_D01G1797	GhCMT1a	D01:55121707-55127592(-)	886	47	AT1G80740	CMT1, DMT4
Gh_A07G0385	GhCMT1b	A07:4924002-4930670(+)	911	48	AT1G80740, AT1G69770	CMT1, CMT3
Gh_D07G0449	GhCMT1c	D07:4841853-4848448(+)	905	48	AT1G80740, AT1G69770	CMT1, CMT3
Gh_A01G1545	GhCMT1d	A01:91977353-91985508(-)	832	43	AT1G80740	CMT1, DMT4
Gh_D02G0626	GhDRM3a	D02:8536542-8541207(-)	702	44	AT3G17310	DRM3, AtDRM3
Gh_A02G0566	GhDRM3b	A02:8572916-8577487(-)	700	43	AT3G17310	DRM3, AtDRM3
Gh_D08G1665	GhCMT2a	D08:52279218-52279355(-)	1,166	48	AT4G19020	CMT2
Gh_A08G1371	GhCMT2b	A08:88191855-88225877(-)	1,154	50	AT4G19020	CMT2
Gh_D04G0527	GhDRM2a	D04:8993428-9007991(+)	640	52	AT5G14620	DRM2, DMT7
Gh_D09G0266	GhDRM2b	D09:8476170-8480695(+)	625	54	AT5G14620	DRM2, DMT7
Gh_A05G3114	GhDRM2c	A05:80312367-80316080(-)	640	51	AT5G14620	DRM2, DMT7
Gh_A09G0264	GhDRM1	A09:8467948-8478422(+)	692	54	AT5G15380	DRM1
Gh_D08G2464	GhDNMT2a	D08:64534904-64538554(+)	384	63	AT5G25480	DNMT2, AtDNMT2
Gh_A08G2094	GhDNMT2b	A08:102185178-102188923(+)	369	66	AT5G25480	DNMT2, AtDNMT2
Gh_A05G3224	GhMET1a	A05:84363661-84370705(-)	1,590	60	AT5G49160	MET1
Gh_D04G0381	GhMET1b	D04:5974938-5981915(+)	1,561	60	AT5G49160	MET1
**DNA demethylases**						
Gh_A04G0555	GhROS1a	A04:35139978-35150131(-)	1,890	67	AT2G36490	DML1, ROS1
Gh_D04G1010	GhROS1b	D04:31885730-31895994(-)	1,893	67	AT2G36490	DML1, ROS1
Gh_A09G2457	GhROS1c	scaffold2307_A09:17856-32958(-)	1,900	55	AT2G36490	DML1, ROS1
Gh_D09G1341	GhROS1d	D09:40650791-40666010(-)	1,949	55	AT2G36490	DML1, ROS1
Gh_A01G1068	GhDML3	A01:39044499-39054377(+)	1,906	48	AT4G34060	DML3
Gh_D01G1149	GhDME	D01:25473810-25483711(+)	1,935	53	AT5G04560	DME

To understand whether DNA methylation plays a role in the development of cotton fruit branching, the expression profiles of cotton DNA methyltransferases and demethylases were analysed. As shown in Fig. 1B, the expression levels of 11 DNA methylation-related genes were changed more than twofold in Gh2nm as compared with those in Gh1nm, and the expression levels of 10 genes were changed more than twofold in Gh3nm as compared with those in Gh1nm. The *GhCMT1* and *GhMET1* DNA methyltransferases, which are the cotton homologs of Arabidopsis *CMT1* and *MET1* and include *GhCMT1a* to *GhCMT1d* and *GhMET1a* and *GhMET1b*, respectively, were notably more expressed in Gh2nm and Gh3nm than in Gh1nm. Among the *DRM2*-homologous genes, *GhDRM2a*-*GhDRM2c* demonstrated a twofold change in expression in Gh2nm and Gh3nm as compared with that in Gh1nm, with the exception of the expression levels of *GhDRM2a* and *GhDRM2c* in Gh3nm as compared with those in Gh1nm. The expression levels of *GhDRM1*, *GhDNMT2a* and *GhDNMT2b* exhibited no obvious differences across the three stages, with the exception of *GhDRM1*, the expression of which was changed more than twofold in Gh3nm compared with that in Gh1nm. Among the DNA demethylases, the expression of *GhROS1a*–*GhROS1d* was no obviously (more than twofold) changed across the three developmental stages of cotton. However, the expression of *GhDML3* and *GhDME*, the cotton homologs of Arabidopsis *DML3* and *DME*, was changed more than twofold in Gh2nm and Gh3nm compared with those in Gh1nm. These data suggest that DNA methylation participates in the development of cotton fruit branches.

### Genomic DNA methylation sequence data

To obtain genomic DNA methylation profiles of fruit branching or floral bud development in cotton, we performed BS-seq for three developmental periods. Approximately 640 million paired-end reads were generated from three samples. After removing low-quality and adapter-polluted reads, the rate of clean reads was above 95%. Unique reads were then mapped to the cotton reference genome sequence. The mapped percentages were 91.63%, 93.44% and 93.23% for Gh1nm–Gh3nm, respectively (Table 2).

**Table 2:** DNA methylation statistics of three cotton developmental periods.

Sample	Gh1nm	Gh2nm	Gh3nm
Raw reads number	642,190,382	633,010,892	639,781,508
Raw bases number	80,273,797,750	79,126,361,500	79,972,688,500
Clean reads number	610,566,542	602,127,016	612,898,844
Clean reads rate (%)	95.08	95.12	95.8
Mapped reads	559,464,906	562,602,893	571,394,177
Mapped ratio (%)	91.63	93.44	93.23
Sequence depth	27.48	27.62	28.05

After the reads were mapped to the cotton reference genome sequence, the cytosine (include CG, CHG, CHH) coverage rates were more than 90% in all three samples (Table S1). The coverage rate for each chromosome was approximately 87–92% (including C, CG, CHG and CHH), and chromosome A08 showed the lowest coverage rate among the three samples (Table S2). For protein-coding gene regions, the coverage rate in CDS (including C, CG, CHG and CHH) was lower than that in introns in all three samples (Table S3). In TE regions, the coverage rate was not obviously different between the three samples, but the coverage rate for multiple TE regions demonstrated certain differences; specifically, the coverage rat in LTR e (approximately 75%) was lower than those in other TE regions (LINE: 93%, SINE: 95%, simple repeat: 89%) in all three samples (Table S4).

Additionally, whole-genome methylation sites (mCs) accounted for 19.73%, 20.48% and 20.18% of total C sequence coverage for the Gh1nm, Gh2nm and Gh3nm samples, respectively. The percentage of mCGs among all CG-covered sequences was 81.14%, 81.35% and 80.9% in the Gh1nm, Gh2nm and Gh3nm samples, respectively. The percentage of mCHG among all CHG coverage was 63.39%, 63.91% and 63.61% in the three samples, respectively, and the percentage of mCHH among all CHH coverage was 4.37%, 5.2% and 4.92%, respectively (Table S5). Further analysis of methylation levels on different chromosomes indicated that chromosomes originating from sub-genome A had methylation levels were higher than those of the corresponding sub-genome D -derived sister chromosomes (for example: methylation levels on chromosome A05 were lower than chromosome D05) (Table S6). Among protein-coding gene regions, CDS methylation levels were lower than those of intronic regions in all three samples (Table S7). mC methylation levels were approximately 4.7% and 8%, while mCG methylation levels were approximately 29% and 55% in CDS and introns, respectively. mCHG and mCHH methylation levels in CDS and introns were also different (approximately 3% and 18% for mCHG and 0.6% and 2% for mCHH, respectively). In TE regions, mC methylation levels first increased and then subsequently decreased following the development of cotton seedlings from Gh1nm to Gh3nm. In every sample, the LTR-type TE methylation levels were higher than those of other TE types (Table S8).

### Distribution of DNA methylation

We identified approximately 100 million mC sites across all three samples (Table S9). Among these mCs, three types (mCG, mCHG, mCHH) were each about 30% in three samples (Gh1nm–Gh3nm) (Table S9; Fig. S2). However, CHH methylation levels were much higher in Gh2nm and Gh3nm than in Gh1nm, and CG methylation levels were lower in Gh2nm and Gh3nm than in Gh1nm.

**Figure 2:**
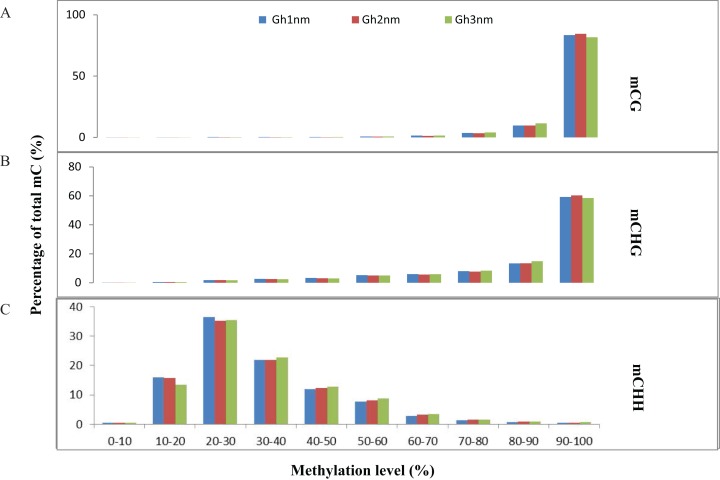
Distribution of the methylation levels in each sequence context for three cotton samples. (A) mCG distribution. (B) mCHG distribution. (C) mCHH distribution. The *x*-axis indicates bins of methylation levels, and the *y*-axis represents the percentage of total methylcytosines in each bin.

In all three cotton samples, the majority of mCG and mCHG site methylation levels were above 90%, whereas mCHH sites exhibited a broader distribution of methylation levels than mCG and mCHG sites (Figs. 2A and 2B). In particular, the majority of mCHH methylation levels were between 10% and 40% (Fig. 2C). Compared with Gh1nm, Gh2nm exhibited increased proportions of highly methylated cytosines in CG and CHG contexts and a slightly decreased percentage of mCs in the CHH context, but Gh3nm showed highly decreased proportions of mCs in CG and CHG contexts and a slightly decreased percentage of mCs in the CHH context compared with Gh1nm (Fig. 2C). In addition, neighbouring (9-bp) °C and mC composition was compared, but there were no notable site-specific differences in CG and CHG composition in the three samples, although CHH was primarily composed of C(A/T/C)(T/A/C), and mCHH was primarily composed of C(T/A/C)(A/T/C) (Fig. S3).

**Figure 3:**
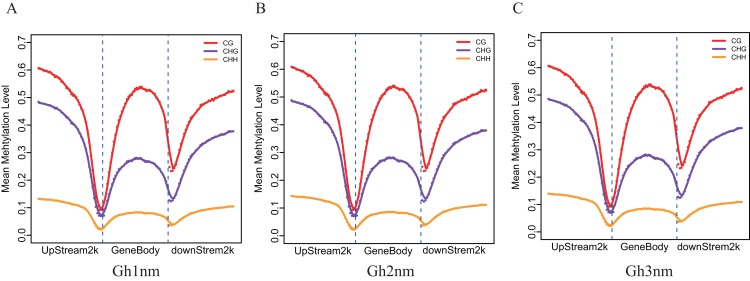
Methylation level distribution of different regions. (A) Gh1nm. (B) Gh2nm. (C) Gh3nm.

To understand DNA methylation patterns in protein-coding genes, methylation profiles of coding genes, including upstream, gene body and downstream flanking regions, were constructed. Levels of two methylation types (mCHG and mCHH) were higher in upstream and downstream flanking regions than in gene body regions, but mCG methylation levels exhibited little difference across all three regions (Figs. 3A–3C).

### Analysis of different methylation regions

When the methylation regions of different samples were compared, DMRs were screened out, and the methylation regions that potentially regulate gene expression were further analysed. Three regions (2000-bp sequence upstream of the coding gene (up2k), the gene body sequence and 2000-bp sequence downstream of the gene body (down2k)) showed differences between the three samples. As shown in Table 3, there were 4,277 CG-type (including 1,141 in up2k, 1,579 in the gene body and 1,557 in down2k), 3,149 CHG-type (including 1,184 in up2k, 1,023 in the gene body and 1,212 in down2k) and 370 CHH-type (including 228 in up2k, 39 in the gene body and 103 in down2k) DMRs that differed between Gh2nm and Gh1nm. Similarly, there were 4,028 CG-type (including 1,082 in up2k, 1,509 in the gene body and 1,437 in down2k), 3,294 CHG-type (including 1,095 in up2k, 1,049 in the gene body and 1,150 in down2k) and 172 CHH-type (including 100 in up2k, 13 in the gene body and 59 in down2k) DMRs that differed between Gh3nm and Gh1nm. Furthermore, there were 5,288 CG-type (including 1,151 in up2k, 2,122 in the gene body and 2,015 in down2k), 4,043 CHG-type (including 1,421 in up2k, 1,220 in the gene body and 1,402 in down2k) and 81 CHH-type (including 46 in up2k, 13 in the gene body and 22 in down2k) DMRs that differed between Gh3nm and Gh2nm. The difference of CG methylation between the three samples occurred mainly in the gene body and downstream sequences, while CHG methylation differences occurred homogeneously in all three regions, and CHH methylation differences occurred primarily in upstream sequences.

**Table 3:** Statistics of the number of DMRs.

	CG			CHG			CHH		
	Up2k	gene	Down2k	Up2k	gene	Down2k	Up2k	gene	Down2k
Gh2nm:Gh1nm	1,169	1,726	1,601	1,166	1,013	1,181	228	38	103
Gh3nm:Gh1nm	1,087	1,655	1,460	1,110	1,020	1,135	104	12	49
Gh3nm:Gh2nm	1,501	2,235	2,051	1,450	1,213	1,402	49	8	28

**Note:**

Up2k means upstream 2,000 bp of gene body; down2k means downstream 2,000 bp of gene body; gene indicates gene body.

DMR-related genes were further analysed by gene ontology (GO). CG- and CHG-type related genes in DMR gene body regions were markedly enriched for Molecular Function when the three samples were compared (Gh2nm vs. Gh1nm, Gh3nm vs. Gh1nm, and Gh3nm vs. Gh1nm) after multiple testing correction (*q*-value ≤ 0.05) (Figs. S4–S6). Markedly enriched GO terms included adenyl, purine binding and transferase activity. When Gh2nm was compared to Gh1nm, CHG-type DMR-related genes were notably enriched for pollen or reproductive processes under the category of Biological Processes.

Kyoto Encyclopedia of Genes and Genomes enrichment analysis of DMR-related genes indicated that CHG-type and CG-type DMR-related genes in Gh2nm and Gh1nm were involved in pathways related to reproduction or floral bud development, such as circadian rhythm (Blumel, Dally & Jung, 2015; Li et al., 2014; Mateos et al., 2015; Qian et al., 2014; Shim & Imaizumi, 2015), alpha-linolenic acid metabolism (Nam & Yoshihara, 2012; Suzuki et al., 2003; Yokoyama et al., 2000) and phosphatidylinositol signaling system (Georges et al., 2009; Ischebeck et al., 2011; Lalanne et al., 2004; Lin et al., 2004; Ma et al., 2004; Potocky et al., 2012).

### The relation between DNA methylation and gene expression

**Figure 4:**
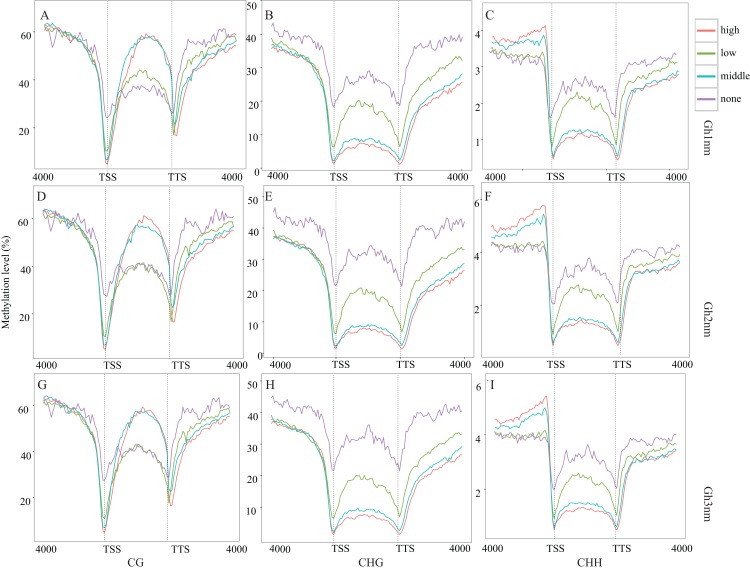
Methylation level of different expression level genes. (A) CG type in Gh1nm; (B) CHG type in Gh1nm; (C) CHH type in Gh1nm; (D) CG type in Gh2nm; (E) CHG type in Gh2nm; (F) CHH type in Gh2nm; (G) CG type in Gh3nm; (H) CHG type in Gh3nm; (I) CHH type in Gh3nm.

The pre-squaring stage (Gh2nm) is a key period for fruit branch growth and floral bud development that influences the transition of Gh1nm from vegetative growth to reproductive growth, and previous GO and KEGG annotation also revealed the enrichment of reproduction-related genes. RNA-seq data for DMRs for all three developmental periods were compared to detect relative changes in methylation and gene expression. The methylation levels of the three types have no notable change (Figs. 3A–3C). Based on our analysis of cotton gene expression levels, there are four gene types (Fig. S7). The methylation levels of these genes were then analysed, and CG-type methylation was close to 60% for the three regions, demonstrating high and medium expression levels. For low-expression and unexpressed genes, methylation levels in upstream and downstream regions were higher than those in gene body. CHG-type methylation levels in upstream and downstream regions for genes characterised by all four different expression levels were higher than those in the gene body, particularly for genes with high and medium expression levels. CHH-type methylation demonstrated a trend similar to that of CHG-type methylation, and methylation levels of the upstream region for high and medium expression genes were significantly higher than those for the gene body and downstream region (Fig. 4A–4I).

An analysis of the DEGs in Gh1nm and Gh2nm revealed 11,743 upregulated genes (including 497 genes expressed only in Gh2nm) and 5,352 downregulated genes (including 164 genes expressed only in Gh1nm) (Fig. S8). Further analysis identified 892 CG-type DMR-related genes, 726 CHG-type DMR-related genes and 84 CHH-type DMR-related genes with differential expression between Gh1nm and Gh2nm (Fig. S9). GO enrichment analysis of the three types of DMR-related DEGs indicated that CG- and CHG-type DEGs were primarily enriched for single-organism processes, metabolic processes and cellular processes under the Biological Processes category and for catalytic activity and binding under the Molecular Function category (Fig. S10). Comparison of Gh1nm with Gh3nm revealed the differential expression of 876 CG-type DMR-related genes, 730 CHG-type DMR-related genes and 42 CHH-type DMR-related genes (Fig. S11).

### The relation between DNA methylation and miRNA expression

**Figure 5:**
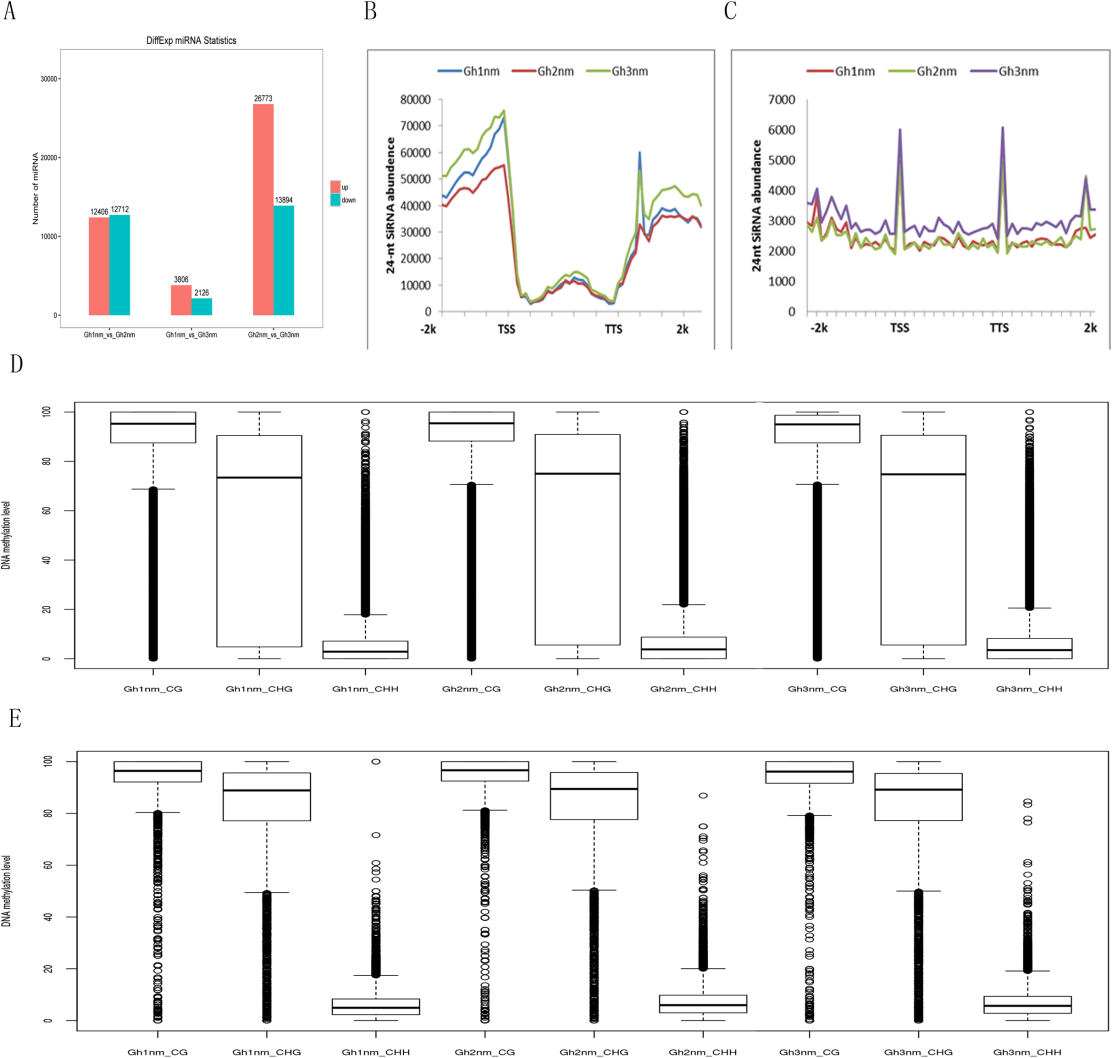
Abundance of 24-nucleotide siRNAs in three stages. (A) The different expressed miRNA number between compared groups. (B) The number of siRNAs distributed in the genebody regions. (C) The number of siRNAs distributed in the TE and flanking 2-kb regions. (D) Methylation levels of three stage in gene body. (E) Methylation levels of three stages in TE.

It has been shown that small RNAs guided de novo DNA methylation at their target loci (Law & Jacobsen, 2010; Mosher & Melnyk, 2010; Xu et al., 2016b). Here, we investigated small RNA expression profiles across all three developmental stages extracted from our previous study (Sun et al., 2016). The 24-nt siRNAs were the most abundant group during all three developmental periods based on the length of their distribution. To analyse the relationship between DNA methylation and small RNA, differently expressed miRNA (DEM) numbers were summed, and the results showed that 12,406 miRNAs were upregulated and 12,712 miRNAs were downregulated in Gh2nm as compared to Gh1nm. When comparing Gh1nm and Gh3nm, 3,806 upregulated and 2,126 downregulated miRNAs were identified, and the comparison of Gh2nm and Gh3nm revealed 26,773 upregulated and 13,894 downregulated miRNAs (Fig. 5A).

In addition, the abundance of 24-nt siRNA in the gene body was lower than that in the upstream and downstream flanking regions. In particular, siRNA abundance in the upstream regions was higher than that in the downstream regions. When we compared the three stages, siRNA abundance in Gh2nm was lower than that in Gh1nm and Gh3nm in the upstream regions (Fig. 5B). We also analysed the siRNA distribution in TE regions and observed a similar trend with higher abundance in upstream and downstream regions, but siRNA abundance in TE regions was lower than that in the gene body (Fig. 5C). Further analysis of 24-nt siRNA abundance in the gene body and TE regions revealed no notable differences in the methylation levels for the three methylation types, with the exception of CHG-type methylation, which was lower in the gene body than in the TE regions. Across all three developmental stages, there were no obvious differences in methylation between the gene body and TE regions (Figs 5D and 5E).

An analysis of the hypermethylation and hypomethylation regions in DMR-related genes containing 24-nt siRNA binding sites revealed no obvious differences in CG-type methylation, but the abundance of hypomethylated CHG-type binding sites in Gh1nm was slightly lower than that in Gh2nm, and hypermethylated CHH-type binding site numbers were higher in Gh1nm than in Gh2nm (Fig. S12). A comparison of Gh1nm and Gh3nm revealed the same trend described above.

### Gene expression profile with siRNA

To characterise the relationship between mRNA, siRNA and DNA methylation, mRNA and siRNA obtained from Huazhong 94-3130 were used to perform correlation analysis between the three developmental stages. There were 131 DEMs with 291 target DEGs between Gh1nm and Gh2nm, 102 DEMs with 217 target DEGs between Gh1nm and Gh3nm, and 64 DEMs with 103 target DEGs between Gh2nm and Gh3nm (Table S10).

**Figure 6:**
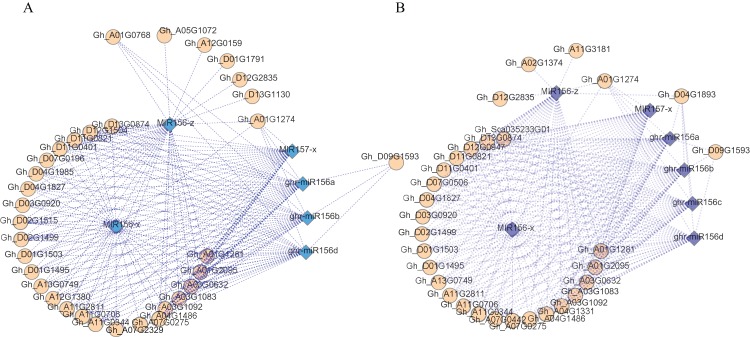
The DEM and corresponding differentially expressed target gene network. (A) The main part of DEM regulatory network between Gh1nm and Gh2nm. (B) The main part of DEM regulatory network between Gh1nm and Gh3nm.

An analysis of the regulatory networks of these DEMs and the corresponding target DEGs indicated that there may be multiple related networks in the compared groups (Gh1nm and Gh2nm, Gh1nm and Gh3nm), primarily including miR156 family members such as miR156a, miR156b, miR156d, miR156x and miR156z (Figs. 6A and 6B). Analysis of the regulated target genes revealed that these miRNA target genes included a series of SPL family homology genes (Table S11).

### Methylation and expression profiles of related genes in the circadian rhythm pathway

**Figure 7:**
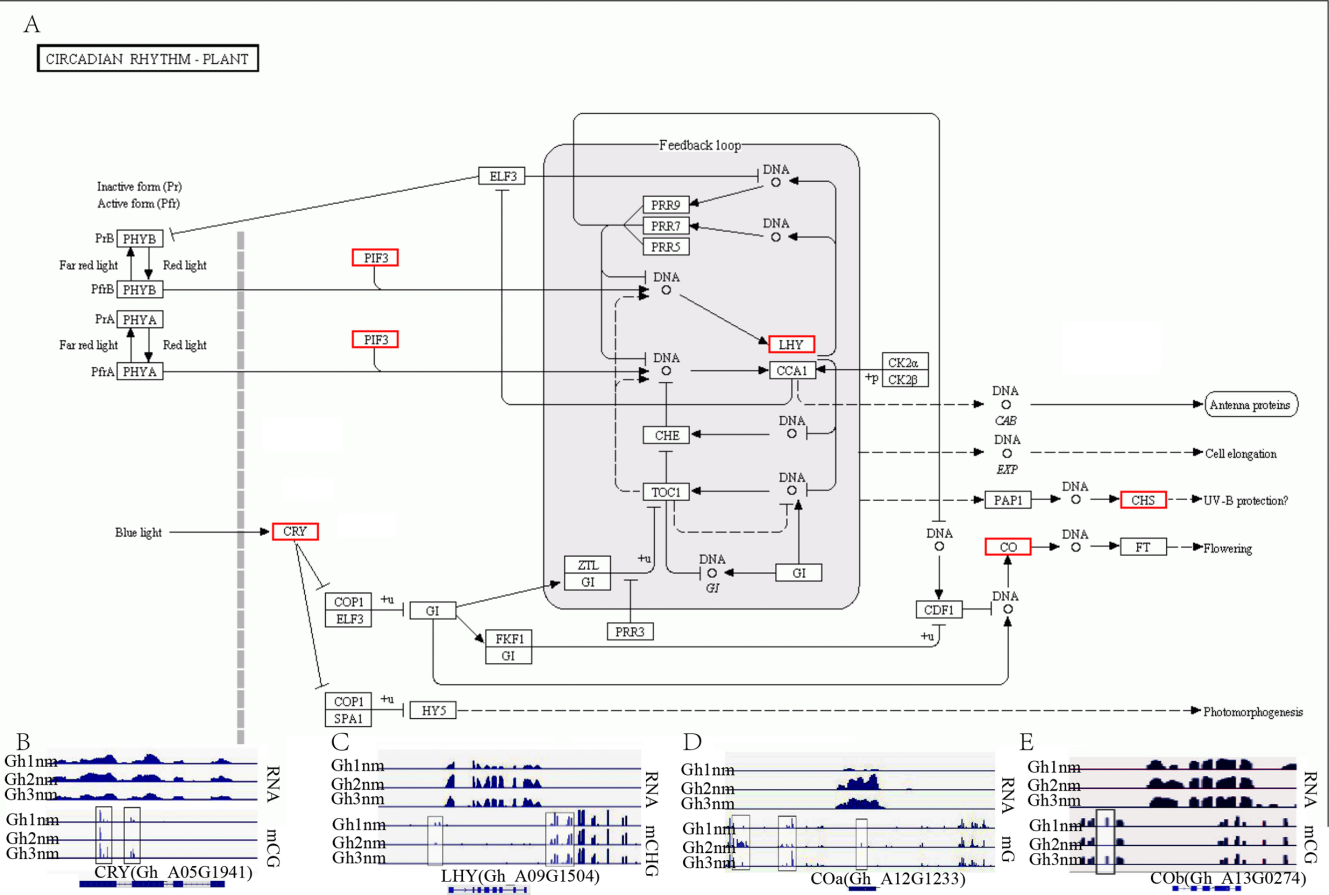
The DNA methylation levels and expression profile of genes in circadian rhythm pathway. (A) The DEM and DGE in circadian rhythm pathway. (B) The DNA methylation levels and gene expression profile of CRY (Gh_A05G1941) at three development stages. (C) The DNA methylation levels and gene expression profile of LHY (Gh_A09G1504) at three development stages. (D–E) The DNA methylation levels and gene expression profile of COa (Gh_A12G1233) and Cob (Gh_A13G0274) at three development stages, respectively. Red box means DEM and DGE genes. Blue box showed different methylation region. RNA (above) showed the expression level and mCG or mC or mCHG (below) showed the methylation profile in (B–E).

In our previous report, we identified multiple genes that were enriched in the circadian rhythm pathway and speculated that this pathway was involved in cotton fruiting branch and floral bud development (Sun et al., 2016). In this study, we further analysed the methylation profiles of DEGs in this pathway and found that the expression levels of CRY, LHY, and CO homology genes exhibited obvious changes across all three developmental stages (Fig. 7A). CRY (Gh_A05G1941) gene expression levels were upregulated in Gh2nm and downregulated in Gh3nm compared to Gh1nm, and CG-type methylation levels of the CRY gene also changed. The methylation levels of the third and last exons decreased in Gh2nm compared with Gh1nm and increased in Gh3nm compared with Gh2nm (Fig. 7B). For the LHY homology gene (Gh_A09G1504), expression levels in Gh2nm and Gh3nm were higher than those in Gh1nm, and CHG-type methylation levels in Gh2nm and Gh3nm were lower than those in Gh1nm (Fig. 7C). COa (Gh_A12G1233) expression in Gh2nm and Gh3nm was significantly higher than that in Gh1nm, and multiple regions of this gene were modified by methylation. COb (Gh_A13G0274) expression levels in Gh2nm were also significantly higher than those in Gh1nm and Gh3nm, and CG methylation levels in the upstream sequence were significantly higher in Gh2nm and Gh3nm than in Gh1nm (Figs. 7D and 7E; Table S12).

**Figure 8:**
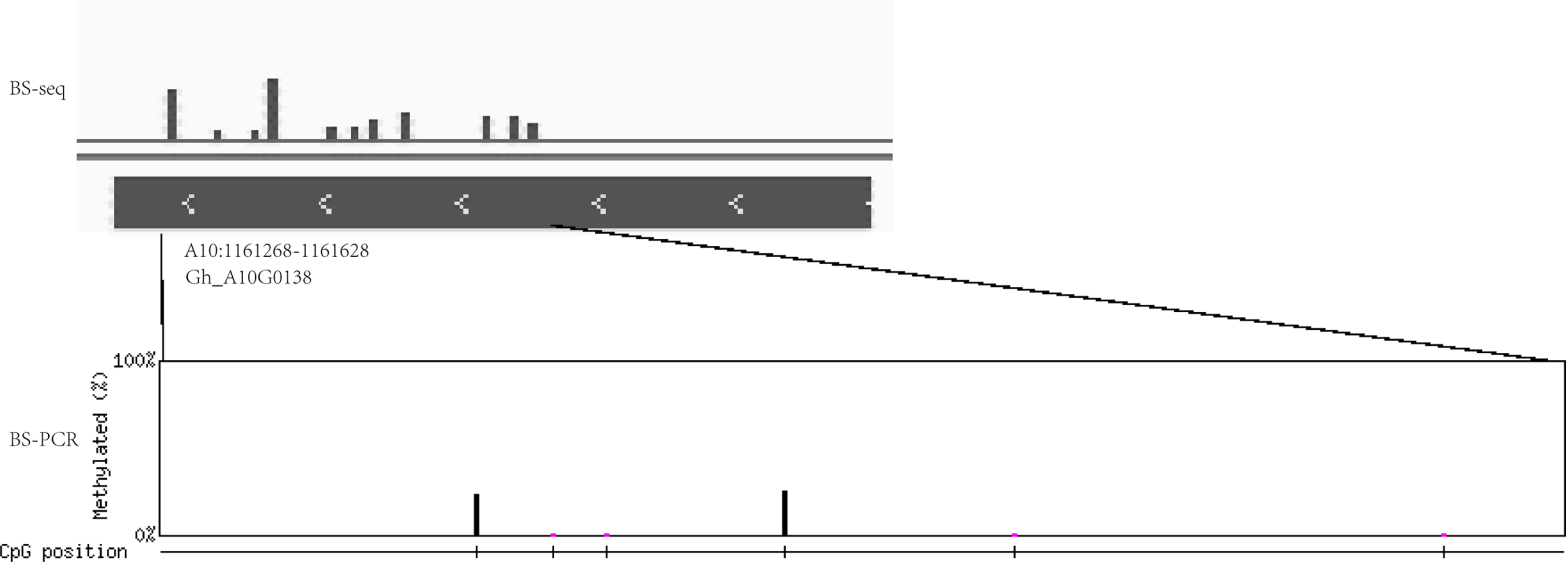
The DNA methylation levels validated by bisulfite PCR sequencing and BS-seq. DNA methylation levels of Gh_A10G0138 was analysed by BS-seq. Vertical bar mean eleven methylation sites with different methylation levels. Methylation site was examined by BS-PCR bisulfite PCR sequencing. Six methylation sites were found. The height of vertical bar means the methylation levels.

In addition, the extent of DNA methylation in Gh_A10G0138 in Gh1nm was experimentally validated by performing bisulfite treatment and clone sequencing, revealing high concordance between the BS-seq data and BS-PCR sequencing results (Fig. 8).

## Discussion

In this study, we globally identified DNA methylation-related sequence regions, investigated the relationship between DNA methylation levels and patterns and mRNA and small RNA expression profiles across the three developmental periods during branching and floral bud formation in cotton. Although the whole-genome BS-seq was performed in a single replicate, multiple plant samples at each stage were pooled together to avoid individual variances. In the data analysis, we also referred to the analytical methods for non-repetitive samples in existing studies and used stringent parameters to reduce deviations. We found that the homologous DNA methyltransferase and demethylase genes in cotton exhibit differential expression during the three developmental stages. In plants, de novo methylation is catalysed by DRM2. In general, CG, CHG and CHH methylations are catalysed by MET1, CMT3 and DRM2, respectively (He, Chen & Zhu, 2011; Law & Jacobsen, 2010). Our data showed that the homologs of *Arabidopsis* CMT1, DRM2 and MET1 were notably upregulated in Gh2nm and Gh3nm as compared to Gh1nm. Although CMT1 function has not been extensively reported, the CMT3 gene primarily mediates CHG-type methylation (Law & Jacobsen, 2010), suggesting that the three types of methylation may be related to the fruiting and flowering of cotton. Among the analysed genes, the expression of four CMT1 homologous genes in Gh2nm and Gh3nm was fourfold more than that in Gh1nm, implying that CHG methylation plays a role in cotton development. Arabidopsis thaliana has one CMT1 gene, while gene-doubling events in tetraploid upland cotton may result in the formation of its four CMT1-homologous genes; however, these four genes were highly expressed in Gh2nm and Gh3nm as compared to Gh1nm, suggesting that additional methylation modification occurs during the latter two developmental stages in cotton. However, whether methyltransferase and demethylase are involved in modifying genes that function in branching and floral bud formation needs further investigation.

During the three developmental stages of cotton, CG-, CHG- and CHH-type methylation levels did not markedly differ (Figs. 3A–3C). Analysis of the methylation levels of DEGs revealed the following features: for highly and moderately expressed genes, CG-type methylation levels in the upstream and downstream regulatory regions changed little relative to the methylation levels in Fig. 3, but CG-type methylation levels in low-expression and unexpressed genes were significantly lower in the gene body region than that in the other regions (Fig. 4). Thus, CG-type methylation modification in the gene body may be related to gene expression and reflect the same trend. For CHG- and CHH-type modifications, methylation levels in regions of genes expressed at high and medium levels were significantly decreased, and methylation and gene expression levels showed an opposite trend. In addition, CHG-type methylation levels in the upstream, gene body and downstream regions of unexpressed genes were higher than that of expressed genes, implying that CHG methylation may be regulated at the expression level. Additionally, methylation levels in the upstream and downstream regions of high and medium-expressed genes were higher than those at low-expressed and unexpressed genes. In general, low methylation levels in upstream gene regions promote gene expression. In this study, we observed a differential relationship for different methylation types: for CG-type methylation, methylation levels in the upstream region were not strongly associated with gene expression level; for CHG-type methylation, upstream methylation levels of expressed genes were lower than those of unexpressed genes, which agrees with the above conclusion; and for CHH-type methylation, upstream methylation levels of genes expressed at high and medium levels were higher than those of low-expression and unexpressed genes, which showed the opposite trend.

The change of the DRM2-homologous gene expression across the three developmental stages may is due to siRNA-mediated methylation modifications. To determine whether methylation modifications affect siRNA expression and control cotton development, the relationship between siRNA and methylation was analysed. As shown in Fig. 5, the DEM between Gh1nm and Gh3nm was smallest, and the abundance of 24-nt siRNA in the upstream and downstream regions of Gh2nm was relatively low compared to Gh1nm and Gh3nm, indicating that Gh2nm miRNAs is involved in unique physiological functions such as the initiation of floral bud or branch formation. Although a large number of 24-nt siRNAs were enriched in the upstream and downstream flanking gene and TE regions, the number associated with upstream and downstream regions was much higher than that associated with TE regions, indicating that siRNA is involved in regulating gene expression. However, the methylation levels of all three types (mCG, mCHG and mCHH) in the 24-nt siRNA binding sites of the upstream and downstream regions were analysed, and there were no significant differences between the three developmental periods; thus, the results did not confirm that methylation affects siRNA binding sites and further regulates gene expression levels. However, siRNA binding site abundance in hypermethylated regions in Gh1nm was higher than that in Gh2nm, indicating that CHH methylation may decrease the siRNA-mediated regulation of gene expression levels.

When we studied the relationship between methylation modifications and cotton branching and bud formation by investigating the GO enrichment of DMR-related genes, we also found that certain genes were enriched in functional groups related to the pollen tube germination (Figs. S4–S6), enhancing our understanding of the relationship between methylation modifications and cotton branch and bud formation. Furthermore, the results were in accordance with our expectations. siRNAs regulate gene expression, and our results from the association analysis of siRNA and gene expression levels revealed a number of differentially expressed miR156 family members. Additionally, we found that their target genes including multiple SPL family members were differentially expressed (Fig. 6). It has been well known that miR156 plays a key role in regulating plant growth, from vegetative growth to reproductive growth, and there are eight miRNA156 family members in Arabidopsis. miR156a and miR156c have dominant roles in controlling flowering timing and increase in their level of histone H3 lysine 27 trimethylation (H3K27me3), and requires this chromatin modification is associated with downregulation of miR156A and miR156C during vegetative phase (Huijser & Schmid, 2011; Jin et al., 2013; Teotia & Tang, 2015; Xu et al., 2016a; Yu et al., 2015). miR156 regulates vegetative growth and reproductive growth in plants primarily through targeting a series of transcription factors such as the SPL gene family (Wang, Czech & Weigel, 2009). Most SPL family genes are target genes of the miR156 family and the miR172 family, which are usually expressed in leaves and shoot apical meristems, and the expressed level are generally negatively correlated. According to our results, multiple miR156 family members and SPL family members were also differentially expressed, indicating that Gh2nm and Gh3nm relative to Gh1nm are the developmental stages controlling floral bud development, which is consistent with the expected physiological development process.

Generally, methylation levels affect gene expression levels, and methylation levels are often negatively correlated with gene expression levels, particularly those in upstream regions. According to our results, although CHG-type methylation levels of highly expressed genes were lower than those of unexpressed genes, there was no strong correlation for other types of methylation and global gene expression, and similar results have been reported for Arabidopsis (Hsieh et al., 2009), rice (He et al., 2010) and castor bean (Xu et al., 2016b). Analysis of the expression and methylation levels of single genes still revealed a negative correlation, but negatively correlated methylation regions were not all concentrated in upstream regulatory sequences and instead were found throughout the gene body and upstream sequence (Fig. 7).

According to a previous study, genes in the circadian rhythm pathway was involved in cotton branch and floral bud development. In the present study, a gene expression profile of the circadian rhythm pathway was constructed and analysed, and many genes were differentially expressed during floral bud development in Huazhong 94-3130 (Fig. S13). Based on further analysis of these DEGs, the methylation levels of multiple homologous genes, including CRY, LHY, and CO, were altered (Fig. 7). However, whether methylation modifications are related to the expression of these genes needs further examination.

## Conclusions

In summary, we identified cotton DNA methylation-related genes and analysed their gene expression profiles. Our data suggest that DNA methylation is involved in cotton floral transition and fruit branching. Although global methylation levels across three developmental stages were not notably different, the CHG-type methylation levels of unexpressed genes were higher than those of expressed genes. In the circadian rhythm pathway, the expression levels of CRY-, LHY- and CO-homologous genes may be associated to DNA methylation changes during different floral bud development, indicating that DNA methylation is involved in branching and flowering. Additionally, whole-genome BS-sequence results indicates that DNA methylation is involved in cotton reproduction and may provide a reference to analyse the molecular mechanisms underlying cotton branching development.

## Supplemental Information

10.7717/peerj.4945/supp-1 Supplemental Information 1 Fig S1. Cartoon representation of selected *Arabidopsis* and *G. hirsutum L.*(Gh) DNA methyltransferases and demethylases. The DNA methyltransferases family, except for DRM3, contain DNA methyltransferase domains (DNA MTase) that have a SCOPd1keaa domain(SCOP); The CMT subfamily contains a DNA MTase, a Chromatin Organization Modifier (CHROMO) and a BAH domain; DRM1 and DRM2 subfamily contains a DNA MTase and two/three Ubiquitin Associated domains (UBA); The DNMT homology genes have one DNA MTase domain; MET1 homology genes contains a DNA MTase and two Bromo-Adjacent Homology domains (BAH); The DNA demethylases family possess endonuclease III (ENDO3c) , a cysteine rich (CXXC) domain, a 4Fe-4S cluster (FES) and a RRM-fold domain present at the C terminus of Demeter-like glycoslyases (DME) and a SCOP domain.

10.7717/peerj.4945/supp-2 Supplemental Information 2 Fig S2. The percentage of methylcytosines of whole reads identified at three developmental stages in cotton.

10.7717/peerj.4945/supp-3 Supplemental Information 3 Fig S3. The site frequency distribution of CG and mCG in whole genome. The round 9bp base of CHG and mCHG(or CHH and mCHH)were used for analysing.

10.7717/peerj.4945/supp-4 Supplemental Information 4 Fig S4. GO enrichment analysis of DMR related genes in compared group Gh2nm and Gh1nm. Red colour means q-value <=0.05.

10.7717/peerj.4945/supp-5 Supplemental Information 5 Fig S5. GO enrichment analysis of DMR related genes in compared group Gh3nm and Gh1nm. Red colour means q-value <=0.05.

10.7717/peerj.4945/supp-6 Supplemental Information 6 Fig S6. GO enrichment analysis of DMR related genes in compared group Gh3nm and Gh2nm. Red colour means q-value <=0.05.

10.7717/peerj.4945/supp-7 Supplemental Information 7 Fig S7. The statistics of expressed level in three periods. None expression (reads per kilobase per million reads mapped(RPKM<=1)), low expression level (1 =100).

10.7717/peerj.4945/supp-8 Supplemental Information 8 Fig S8. The differentially expressed genes(DGEs) statistics between Gh1nm and Gh2nm. Up gene: up-regulation gene in Gh2nm as compared to Gh1nm. Special up gene: up-regulation gene in Gh2nm as compared to Gh1nm and no expressed in Gh1nm. down gene: down-regulation gene in Gh2nm as compared to Gh1nm. Special down gene: down-regulation gene in Gh2nm as compared to Gh1nm and no expressed in Gh2nm.

10.7717/peerj.4945/supp-9 Supplemental Information 9 Fig S9. Comparison of DGEs according to DMRs related genes between Gh1nm and Gh2nm. Number showed the total gene counts in the respective group.

10.7717/peerj.4945/supp-10 Supplemental Information 10 Fig S10. The GO analysis of the three type DMRs related genes between Gh1nm and Gh2nm.

10.7717/peerj.4945/supp-11 Supplemental Information 11 Fig S11. Comparison of DGEs according to DMRs related genes between Gh1nm and Gh3nm. Number showed the total gene counts in the respective group.

10.7717/peerj.4945/supp-12 Supplemental Information 12 Fig S12. Abundance of 24-nt siRNAs located to the three type hypermethylated and hypomethylated regions for DMRs related genes between the compared groups Gh1nm: Gh2nm and Gh1nm: Gh3nm.

10.7717/peerj.4945/supp-13 Supplemental Information 13 Fig S13. DEGs enriched in circadian rhythm pathway between the compared groups Gh1nm: Gh2nm.

10.7717/peerj.4945/supp-14 Supplemental Information 14 Table S1. The coverage of cytosines in three cotton development period.

10.7717/peerj.4945/supp-15 Supplemental Information 15 Table S2. The coverage ratio of cytosines at the chromosome level in three cotton development period.

10.7717/peerj.4945/supp-16 Supplemental Information 16 Table S3. The coverage ratio of cytosines at protein coding gene region in three cotton development period.

10.7717/peerj.4945/supp-17 Supplemental Information 17 Table S4. The coverage ration of cytosines at the repeat region in three cotton development period.

10.7717/peerj.4945/supp-18 Supplemental Information 18 Table S5. The Methylation ratio of the cotton whole genome.

10.7717/peerj.4945/supp-19 Supplemental Information 19 Table S6. The Methylation level of cytosines at the chromosome level in three cotton development period.

10.7717/peerj.4945/supp-20 Supplemental Information 20 Table S7. The Methylation ratio of protein-coding gene region.

10.7717/peerj.4945/supp-21 Supplemental Information 21 Table S8. The Methylation level of cytosines at the repeat region in three cotton development period.

10.7717/peerj.4945/supp-22 Supplemental Information 22 Table S9. The number of different type mC in three samples.

10.7717/peerj.4945/supp-23 Supplemental Information 23 Table S10. Statistics of DEM related different expression target gene.

10.7717/peerj.4945/supp-24 Supplemental Information 24 Table S11. The DEM target gene annotation.

10.7717/peerj.4945/supp-25 Supplemental Information 25 Table S12. The DEM methylation states and related DEG expression level compared Gh1nm and Gh2nm.

## Additional Information and Declarations

### Data Availability

The following information was supplied regarding data availability: SRR5137778, SRR5137779, SRR5137780, SRP095905.
