# Supplementary figures and images for: Changes in DNA methylation assessed by genomic bisulfite sequencing suggest a role for DNA methylation in cotton fruiting branch development
Source: PeerJ. 2018 Jun 14;6:e4945. doi: 10.7717/peerj.4945 (PMC6004305; doi:10.7717/peerj.4945)

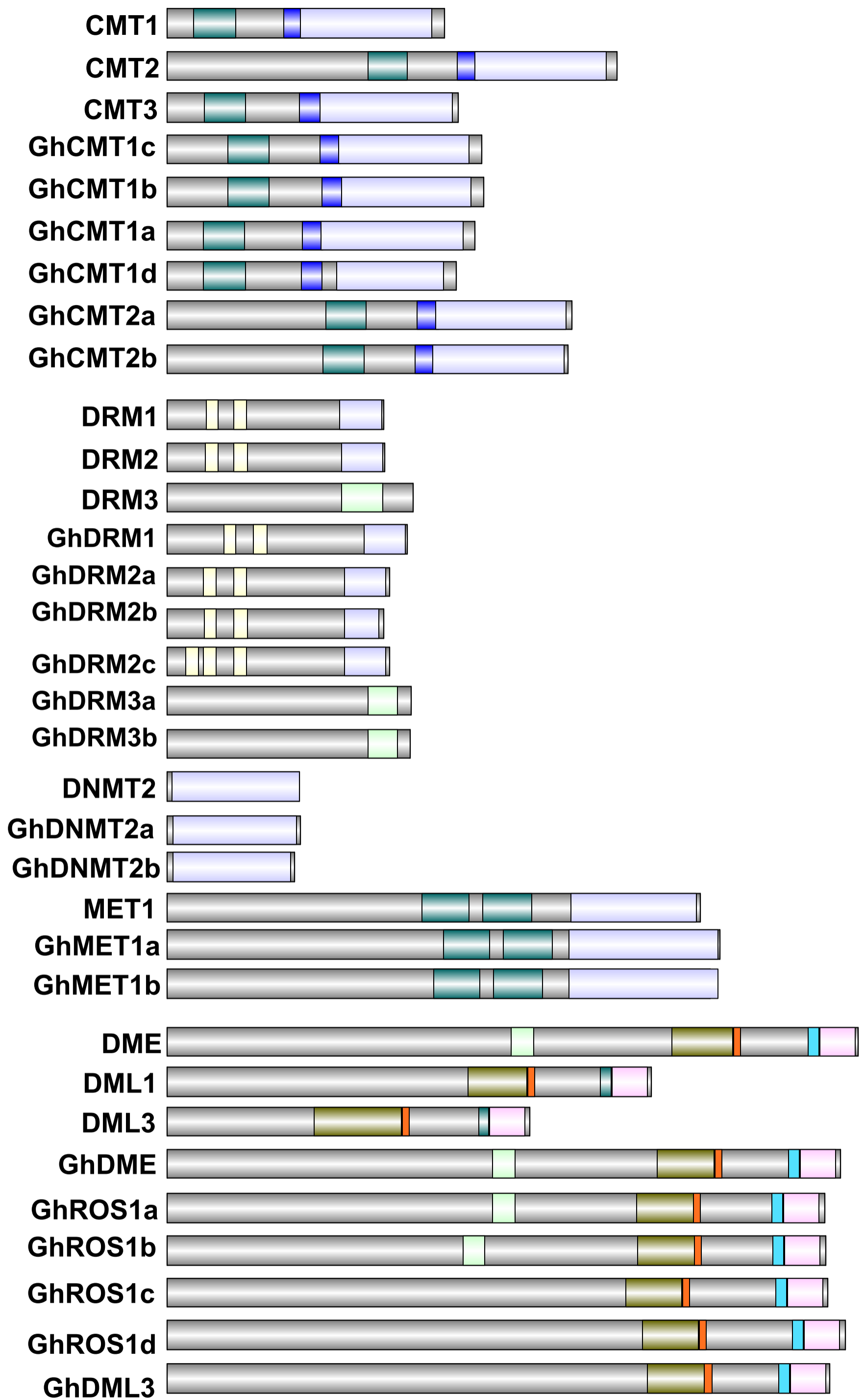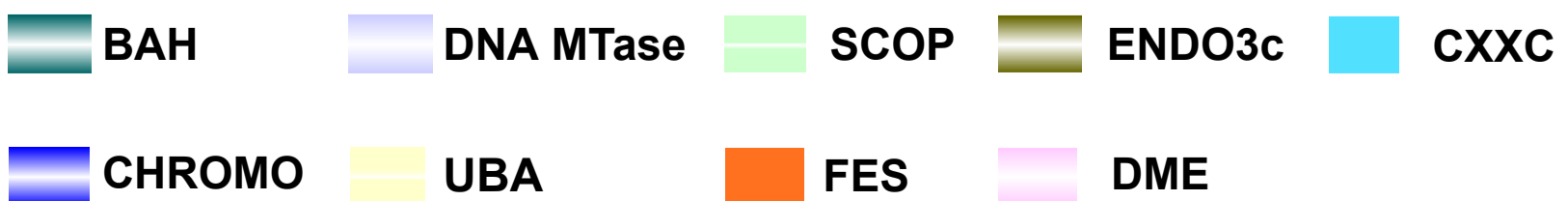

Supplement: Supplemental Information 1 — The DNA methyltransferases family, except for DRM3, contain DNA methyltransferase domains (DNA MTase) that have a SCOPd1keaa domain(SCOP); The CMT subfamily contains a DNA MTase, a Chromatin Organization Modifier (CHROMO) and a BAH domain; DRM1 and DRM2 subfamily contains a DNA MTase and two/three Ubiquitin Associated domains (UBA); The DNMT homology genes have one DNA MTase domain; MET1 homology genes contains a DNA MTase and two Bromo-Adjacent Homology domains (BAH); The DNA demethylases family possess endonuclease III (ENDO3c) , a cysteine rich (CXXC) domain, a 4Fe-4S cluster (FES) and a RRM-fold domain present at the C terminus of Demeter-like glycoslyases (DME) and a SCOP domain. [file peerj-06-4945-s001.pdf]

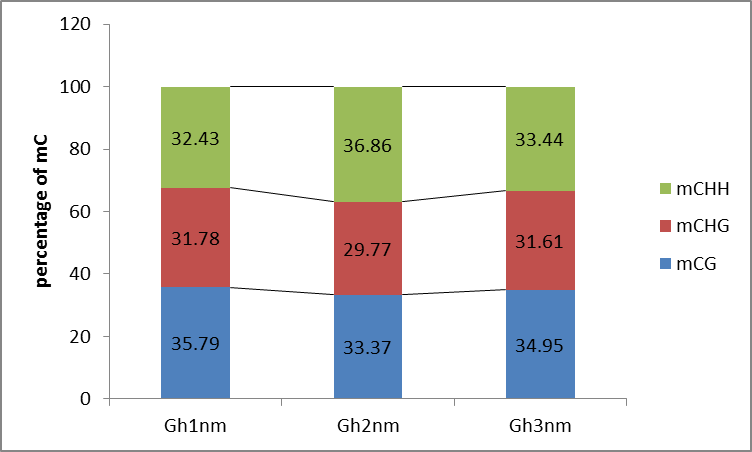

Supplement: Supplemental Information 2 [file peerj-06-4945-s002.png]

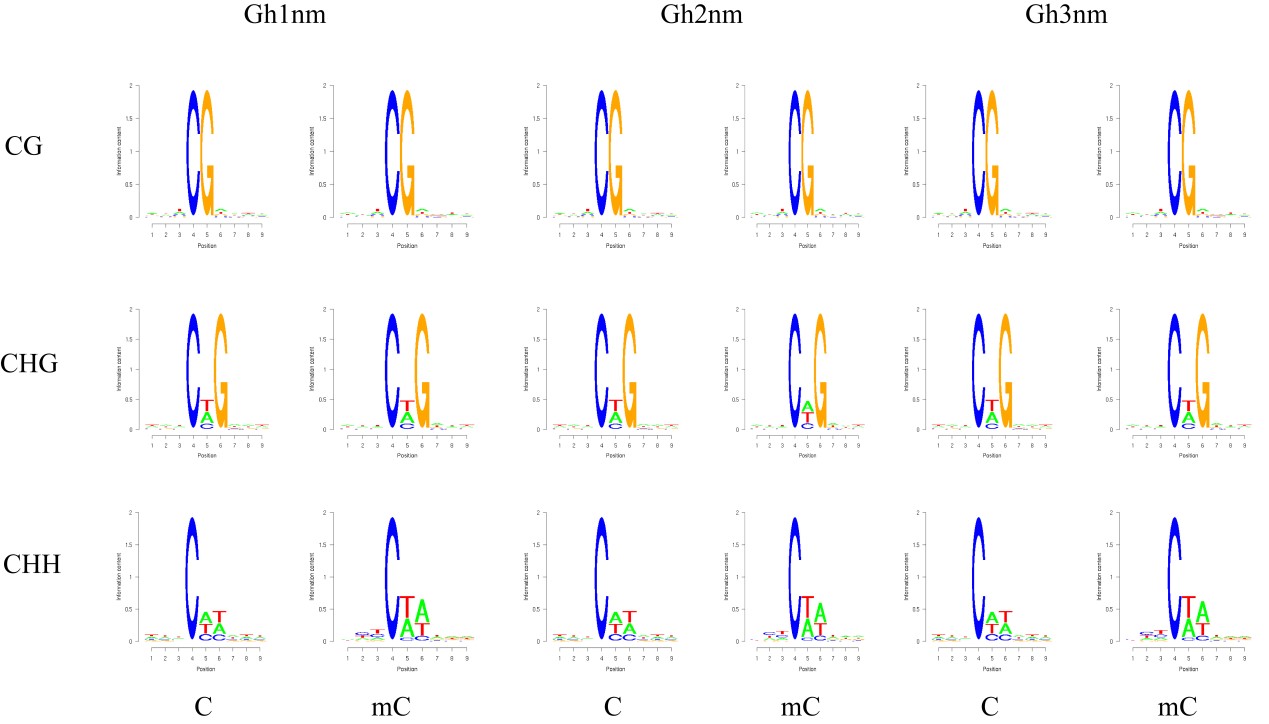

Supplement: Supplemental Information 3 — The round 9bp base of CHG and mCHG(or CHH and mCHH)were used for analysing. [file peerj-06-4945-s003.jpg]

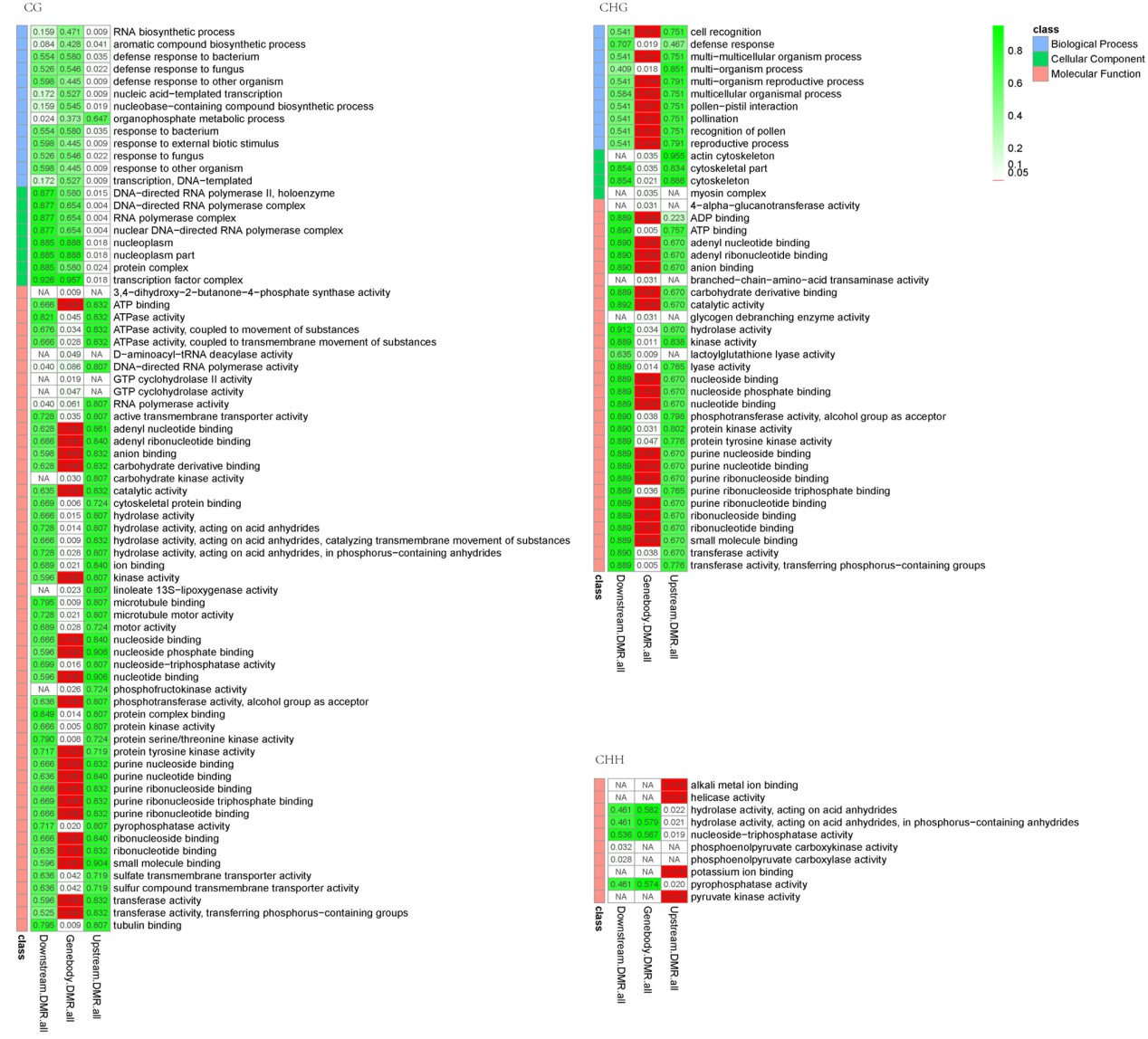

Supplement: Supplemental Information 4 — Red colour means q-value <=0.05. [file peerj-06-4945-s004.jpg]

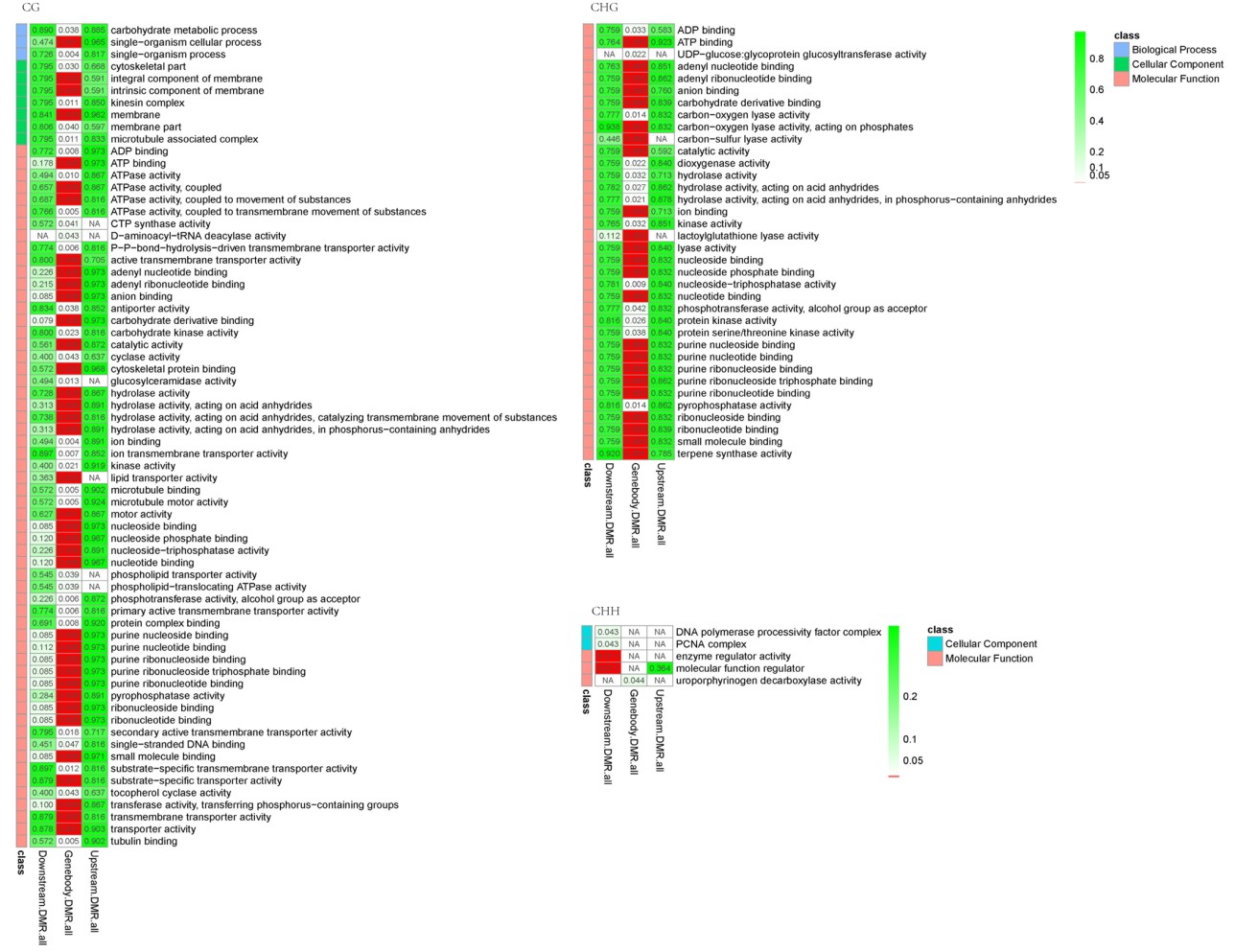

Supplement: Supplemental Information 5 — Red colour means q-value <=0.05. [file peerj-06-4945-s005.jpg]

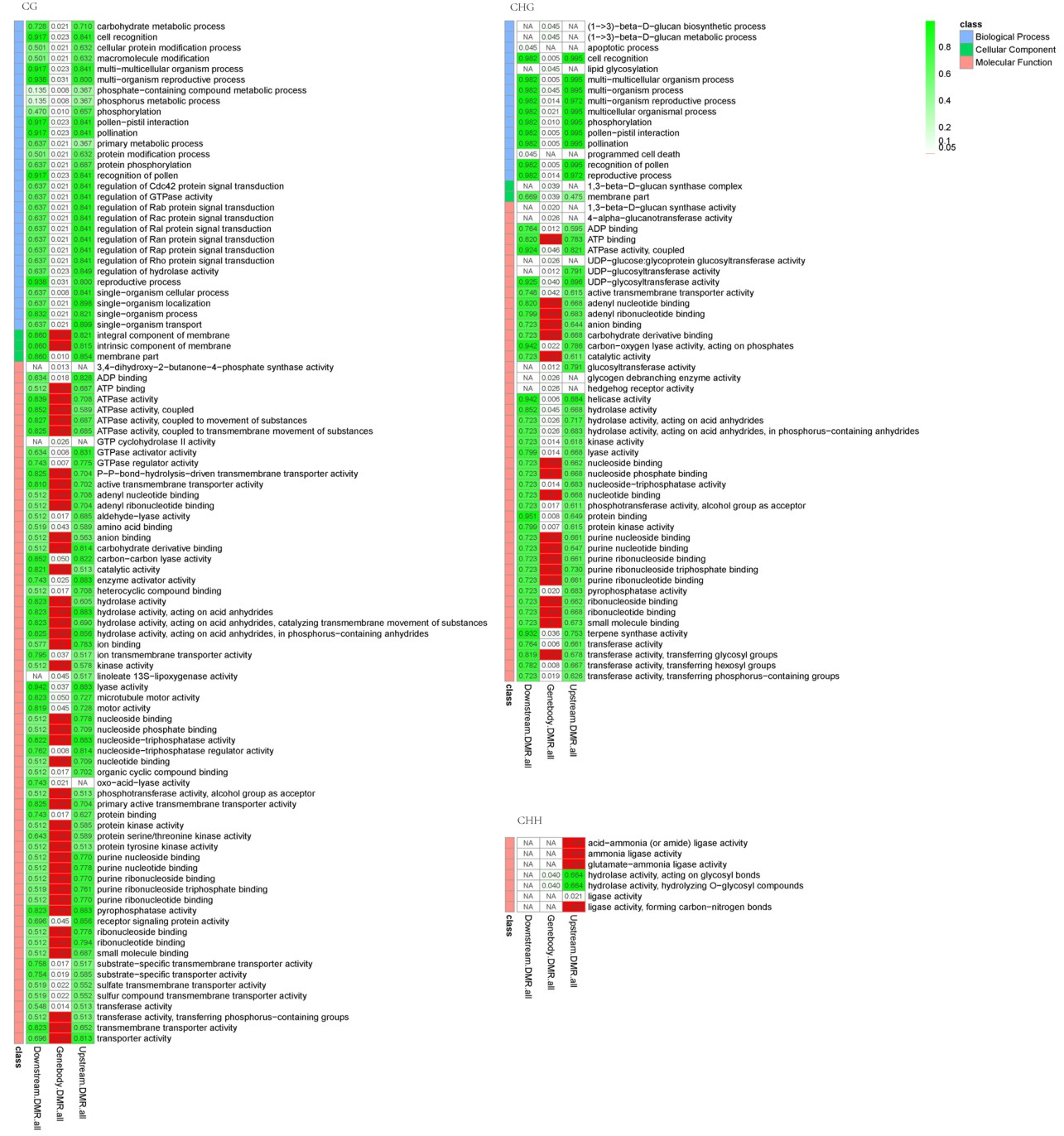

Supplement: Supplemental Information 6 — Red colour means q-value <=0.05. [file peerj-06-4945-s006.jpg]

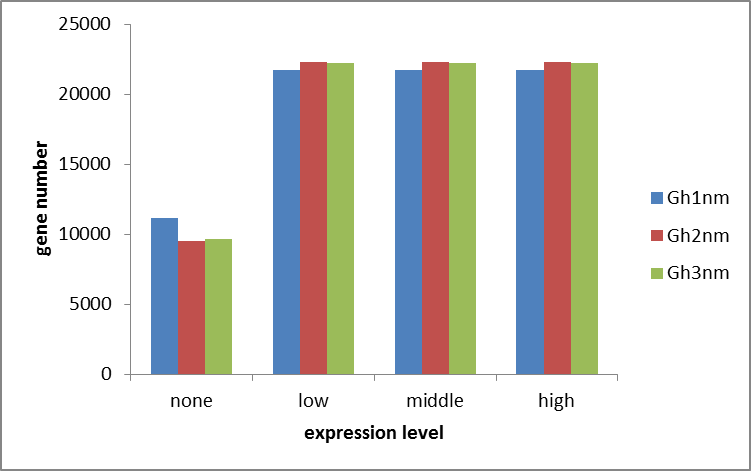

Supplement: Supplemental Information 7 — None expression (reads per kilobase per million reads mapped(RPKM<=1)), low expression level (1 =100). [file peerj-06-4945-s007.png]

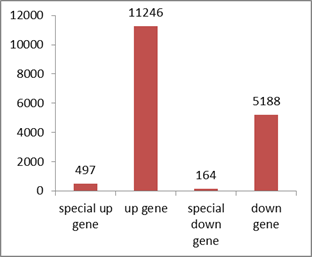

Supplement: Supplemental Information 8 — Up gene: up-regulation gene in Gh2nm as compared to Gh1nm. Special up gene: up-regulation gene in Gh2nm as compared to Gh1nm and no expressed in Gh1nm. down gene: down-regulation gene in Gh2nm as compared to Gh1nm. Special down gene: down-regulation gene in Gh2nm as compared to Gh1nm and no expressed in Gh2nm. [file peerj-06-4945-s008.png]

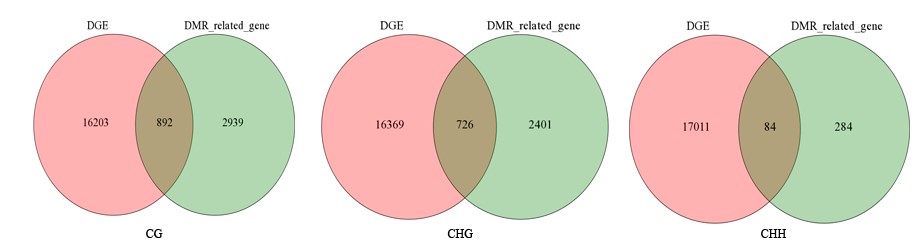

Supplement: Supplemental Information 9 — Number showed the total gene counts in the respective group. [file peerj-06-4945-s009.jpg]

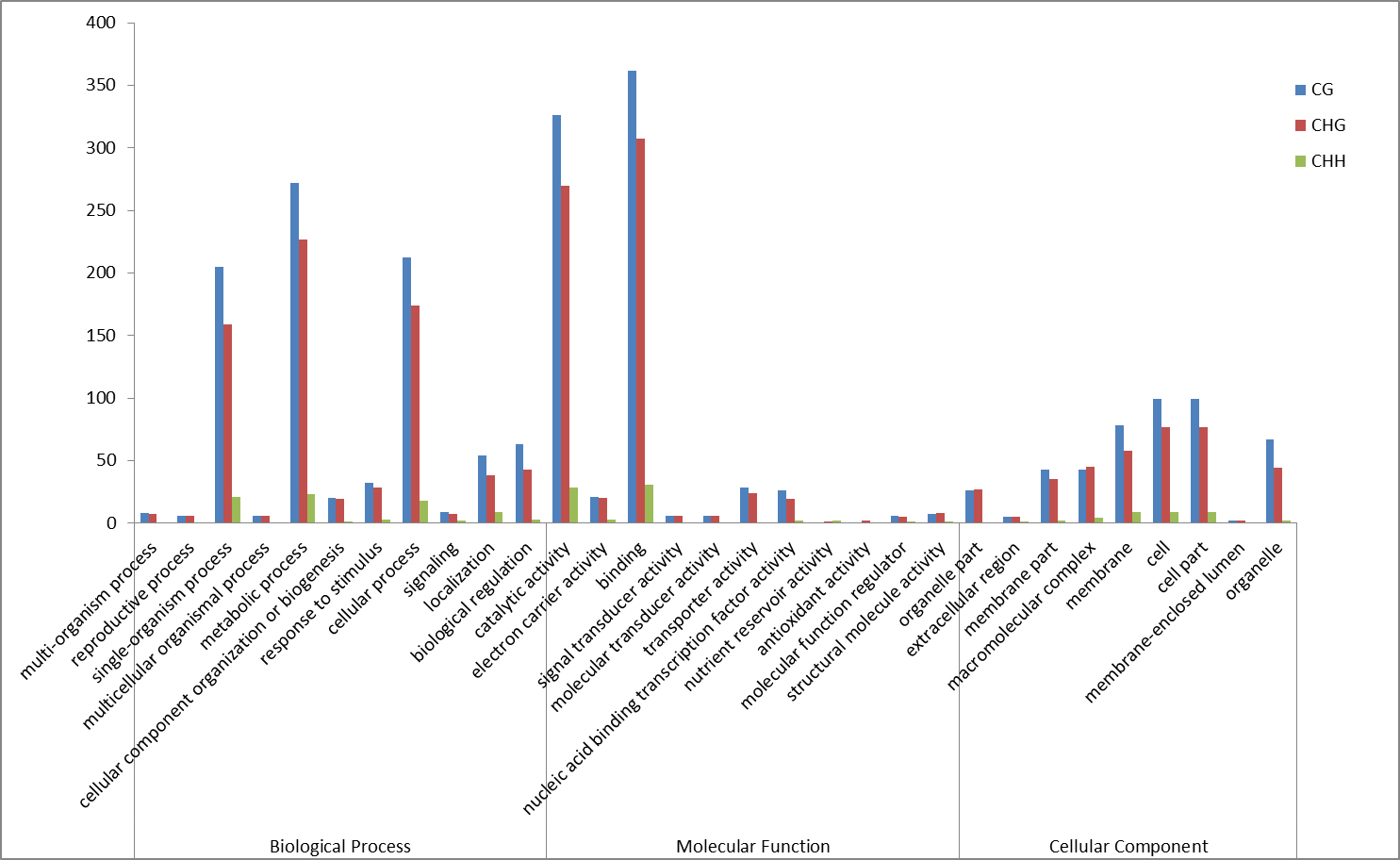

Supplement: Supplemental Information 10 [file peerj-06-4945-s010.jpg]

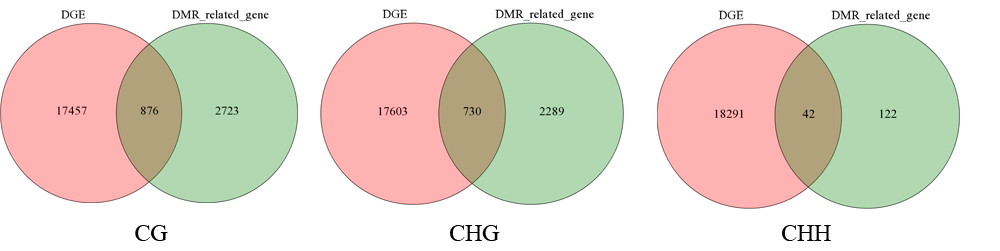

Supplement: Supplemental Information 11 — Number showed the total gene counts in the respective group. [file peerj-06-4945-s011.png]

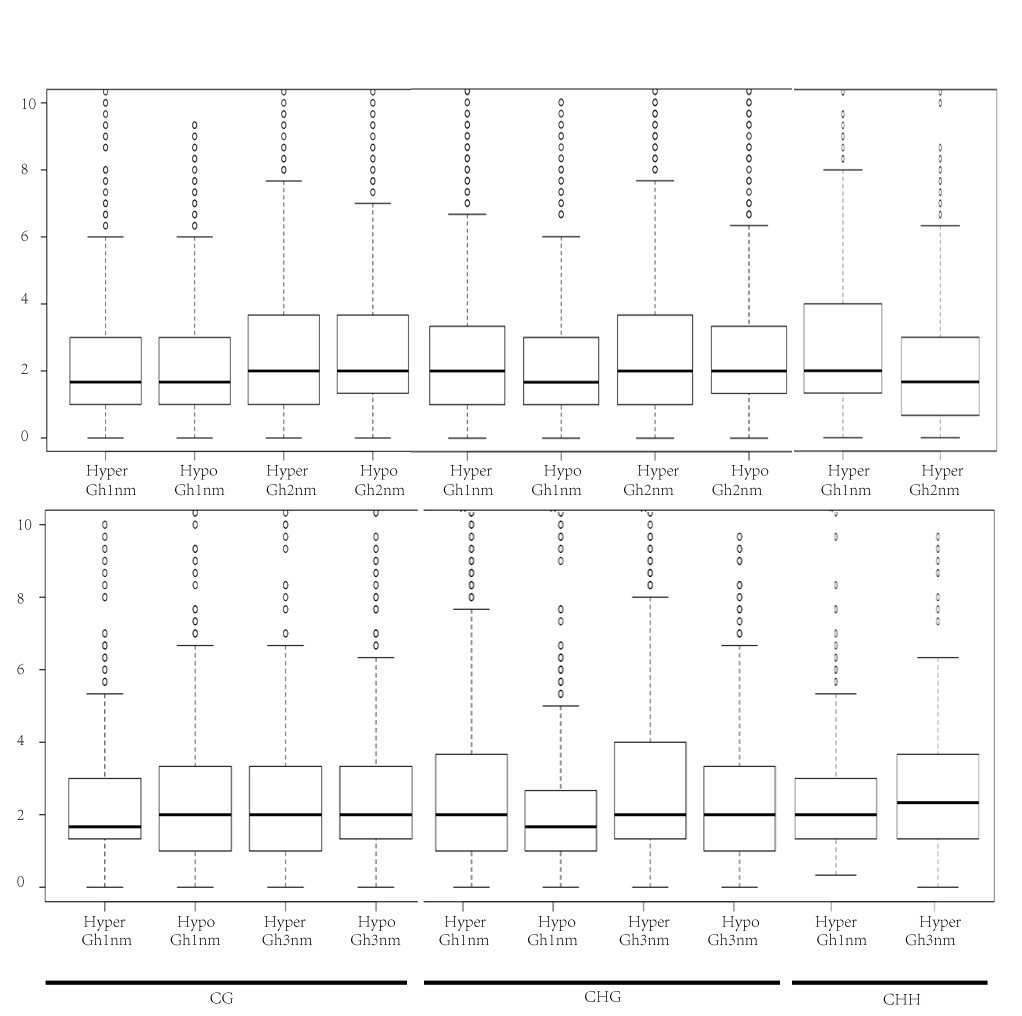

Supplement: Supplemental Information 12 [file peerj-06-4945-s012.jpg]

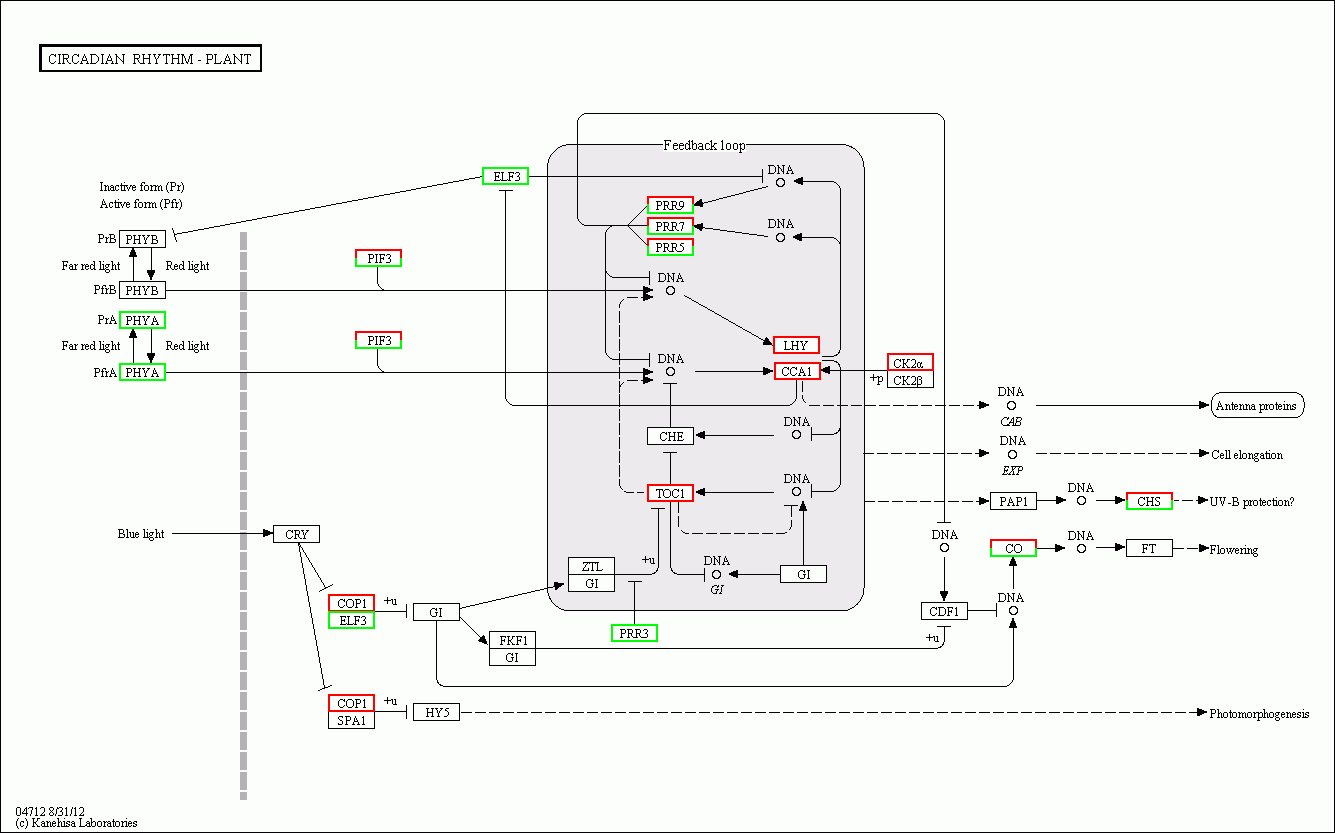

Supplement: Supplemental Information 13 [file peerj-06-4945-s013.png]
